# Proactive Construction of an Annotated Imaging Database for Artificial Intelligence Training

**DOI:** 10.1007/s10278-020-00384-4

**Published:** 2020-11-09

**Authors:** Caroline Bivik Stadler, Martin Lindvall, Claes Lundström, Anna Bodén, Karin Lindman, Jeronimo Rose, Darren Treanor, Johan Blomma, Karin Stacke, Nicolas Pinchaud, Martin Hedlund, Filip Landgren, Mischa Woisetschläger, Daniel Forsberg

**Affiliations:** 1 Center for Medical Image Science and Visualization (CMIV), Linköping University Hospital, Linköping University, SE-581 85 Linköping, Sweden; 2 Department of Health, Medicine and Caring Sciences (HMV), Linköping University, SE-581 85 Linköping, Sweden; 3 Sectra AB, Teknikringen 20, SE-583 30 Linköping, Sweden; 4 Department of Science and Technology (ITN), Linköping University, Campus Norrköping, SE-601 74 Norrköping, Sweden; 5 Department of Clinical Pathology, Region Östergötland, Linköping University Hospital, SE-581 85 Linköping, Sweden; 6 Department of Biomedical and Clinical Sciences (BKV), Linköping University, SE-581 85 Linköping, Sweden; 7 Department of Cellular Pathology, Leeds Teaching Hospital NHS Trust, Beckett St, Leeds, LS9 7TF UK; 8 University of Leeds, Leeds, LS2 9JT UK; 9 Department of Radiology, Region Östergötland, Linköping University Hospital, SE-581 85 Linköping, Sweden; 10 ContextVision AB, Klara Norra Kyrkogata 31, SE-111 22 Stockholm, Sweden

**Keywords:** Artificial intelligence, Annotation, Case collection, Radiology, Pathology

## Abstract

Artificial intelligence (AI) holds much promise for enabling highly desired imaging diagnostics improvements. One of the most limiting bottlenecks for the development of useful clinical-grade AI models is the lack of training data. One aspect is the large amount of cases needed and another is the necessity of high-quality ground truth annotation. The aim of the project was to establish and describe the construction of a database with substantial amounts of detail-annotated oncology imaging data from pathology and radiology. A specific objective was to be proactive, that is, to support undefined subsequent AI training across a wide range of tasks, such as detection, quantification, segmentation, and classification, which puts particular focus on the quality and generality of the annotations. The main outcome of this project was the database as such, with a collection of labeled image data from breast, ovary, skin, colon, skeleton, and liver. In addition, this effort also served as an exploration of best practices for further scalability of high-quality image collections, and a main contribution of the study was generic lessons learned regarding how to successfully organize efforts to construct medical imaging databases for AI training, summarized as eight guiding principles covering team, process, and execution aspects.

## Introduction

Globally, a constantly increasing workload and cost causes an extremely demanding situation for both the radiology and pathology disciplines. However, the rapid development of artificial intelligence (AI) technology shows great potential for highly desired improvements. Future tools are expected to contribute to improving the quality, accuracy, and efficiency of diagnostic work and would be of particular use for repetitive and time-consuming tasks.

A prerequisite for successful machine learning development in imaging diagnostics is the availability of large volumes of labeled (annotated) data. Today, there are some existing repositories available, for example: The Cancer Imaging Archive [1], re3data [2], Grand Challenges in Biomedical Image Analysis [3], Chest-Xray8 [4], MIMIC-CXR Database [5], and Image Collection Library for Histopathology Image Processing [6]. However, the demand for labeled data is immense, and lack of suitable annotated data is considered a major bottleneck for AI development in the field of medical imaging. The lack pertains to several aspects: amount of cases, coverage of relevant diagnostic areas, and quality/existence of annotations [7]. With respect to ground truth annotations, inconsistencies in labeling have been shown to be problematic [8]. Moreover, since AI models need to be continuously updated as the underlying imaging techniques evolve, there will be a persistent need for more training data even for already trained and proven models. Therefore, it is of utmost importance to develop sustainable processes and practices for generation of high-quality annotated imaging data. An important facet of high quality is representativeness for clinical tasks, both in terms of pixel data and annotated components.

In this paper, we describe the construction of an annotated imaging database including data from both radiology and pathology. The database construction was proactive in the sense that the intent was to support future AI training across a wide range of tasks. The proactiveness entails challenges of generality, which do not emerge in annotation projects serving a predefined and highly specific AI training target. The construction of the database constitutes an initial phase that was intended to explore best practices for further work on a larger scale. We argue that a broad knowledge on organization and workflow aspects of database construction is of a great value for AI training in medical imaging. Few such insights have previously been reported in the literature, and the focus for those studies has mainly been to describe the database content [9, 10]. Thus, apart from the database as such, our contribution is lessons learned from its generation, summarized in eight guiding principles covering team, process, and execution facets. While our work targets proactive database design and construction, we believe that these guiding principles are valid in many other cases including specifically targeted annotation efforts.

## Materials and Methods

The work presented in this paper was conducted within a project called DROID, the Diagnostic Reference Oncology Imaging Database. The project was run by a triple-helix consortium consisting of an academic institution (CMIV, Linköping University), the diagnostic division of one public care provider (Region Östergötland), and two companies working in AI for medical imaging (Sectra and Context Vision).

The aim of the project was to establish a database with substantial amounts of detail-annotated oncology imaging data from pathology and radiology, as a first step towards developing high-quality collections on a large scale. The database generation was proactive, in the sense that it was intended to be useful for unspecified future training of AI models for many different assistance tasks, such as detection, quantification, segmentation, and classification.

This study was approved by the regional institutional review board prior to study commencement (2017-276-31 and 2017-285-31). At the onset of the project, clinically active physicians evaluated which medical areas within oncology to target. The first selection criterion was clinical relevance in terms of demand for AI assistance, feasibility of an AI tool to successfully contribute, and feasibility to generate sufficient image data to train an AI model. The second selection criterion was diversity in terms of the tasks requested from AI assistance, such as detection or segmentation. In pathology, four areas were selected, breast, ovary, skin, and colon, and, in radiology, two areas, skeleton and liver.

The annotation work was made by medical experts in the respective field, carefully taking standardized ontologies into account. In parallel to annotation work based on traditional polygon drawing, prototypes of annotation tools with tailor-made interaction design and machine learning assistance were developed and evaluated [11]. For each of the four pathology datasets, one physician made the annotations and, thenceforth, one senior pathologist confirmed them. In total, two senior pathologists and one other physician were involved in the project. For each of the two radiology datasets, one radiology resident was responsible for making the annotations and one senior radiologist confirmed them. In total, one senior radiologist and two radiology residents were involved in the project.

The annotation effort was conducted in combination with AI prototype development using the data, constituting a usage-centered feedback loop allowing continuous improvements during the database creation. The purpose was to give early feedback on the annotation protocol design and to investigate the utility of the database. Three AI models were developed, two for pathology and one for radiology.

## Results

### Database Content

The DROID project resulted in a database containing images with detailed annotations for training of AI systems for oncology diagnostics. The database covers both the area of pathology and radiology, and the content is well diversified. Malignant as well as benign images of liver, skeleton, skin, colon, breast, and ovary can be accessed for medical AI training proposes. This includes 754 pathology whole-slide images and 110 CT examinations (Table 1).

**Table 1:** Diagnostic reference oncology imaging database (DROID) content

Pathology WSI			
Tissue	Images	Annotations	Size (GB)
Ovarian	193	2402	109
Breast	361	4144	501
Skin	99	16,741	32
Colon	101	756	49
Total	754	24,043	691
Radiology CT			
Tissue	Cases	Lesions	Size (GB)
Abdomen (liver)	76	361	34
Skeleton (pelvis)	34	36	15
Total	110	397	49

The image database has initially been published for open use within Sweden through AIDA, Analytic Imaging Diagnostics Arena [12], a national arena for AI research and innovation. The AIDA dataset register [13] is open for exploration also by international researchers, and access requests are welcome.

The datasets are briefly described below. All imaging data were anonymized prior to further handling. The data were mainly collected from the clinical PACS at the Linköping University Hospital, from the Pathology and Radiology departments, respectively, apart from the breast histopathology that was scanned from a separate research collection of slides. An initial subset of the colon and skin datasets have been described previously [14]. The annotations were made in the Sectra PACS software (Sectra AB, Linköping, Sweden). For three of the datasets (breast, ovary, and liver), a corresponding AI prototype was developed in parallel. For further specification of each DROID dataset, please refer to the Appendix.

### Histopathology—Ovarian Tissue

**Fig. 1:**
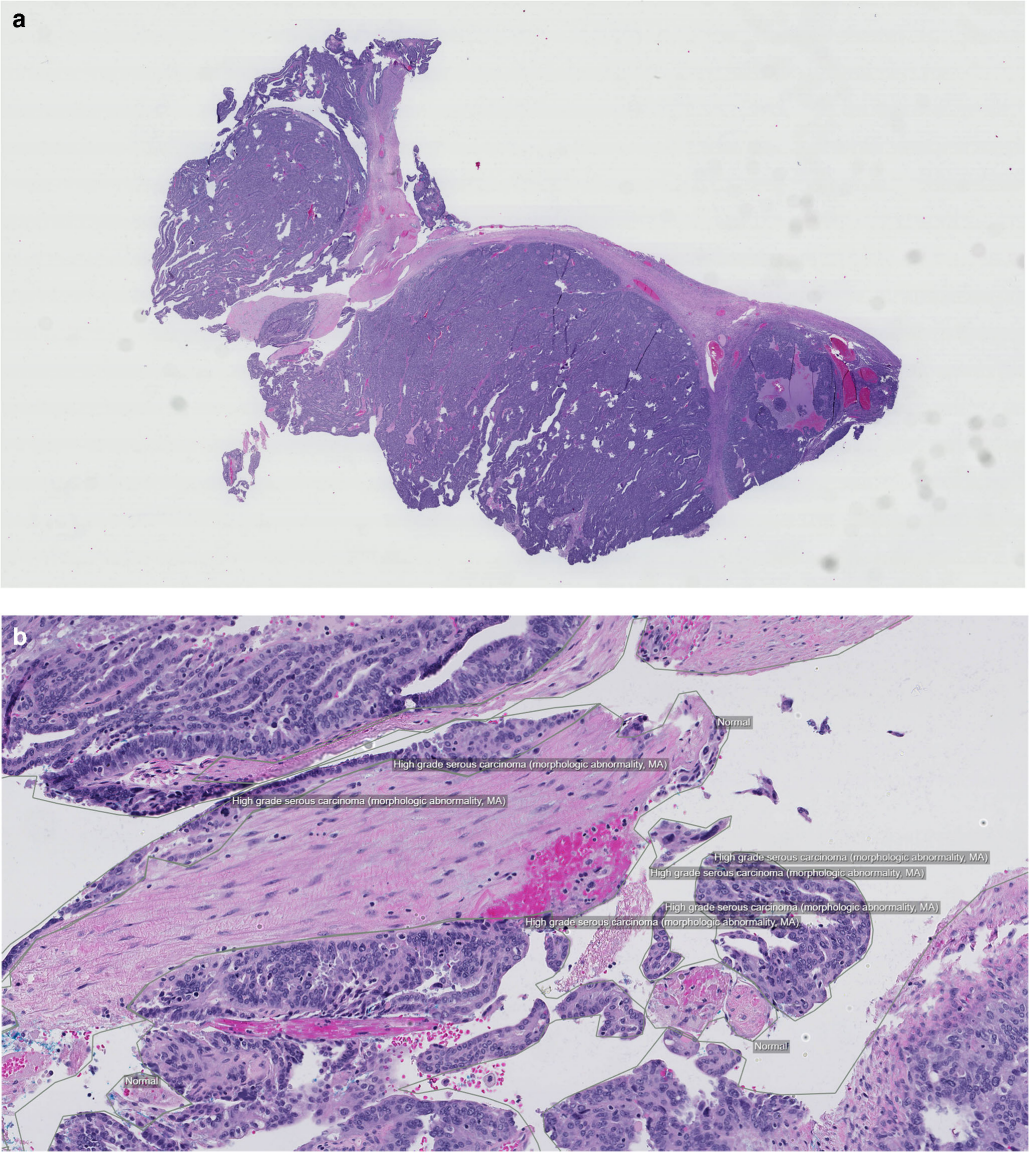
Example images from the DROID ovarian histopathology dataset selected to reflect the overall distribution of clinical cases of suspected ovarian tumors. (**a**) Full-slide overview. (**b**) Close-up showing annotated regions

The ovarian dataset [15] consists of 193 hematoxylin and eosin (H&E)-stained whole slide images (WSI) (Fig. 1). The distribution of diagnostic classifications was selected to reflect the overall distribution of clinical cases of suspected ovarian tumors. The dataset consists of 172 malignant tumor cases and 21 normal cases. Eight of the most prevalent, histologically defined, tumor types were annotated by polygonal outlines. Normal ovarian tissue was outlined as a whole. In total, 2402 separate annotations were made.

The corresponding developed AI prototype targeted the prediction of whether additional immunohistochemistry (IHC) staining is needed for clinical diagnosis. High-grade serous carcinoma (HGSC) is the most common type of cancer originating from the ovary/tuba, characterized by low differentiated cells with a high mitosis rate. The H&E stain is, however, insufficient to set the HGSC diagnosis as other carcinoma types can have a similar morphology. If any of these cancer types are found, the clinical process is to order additional tissue sections stained by certain IHC markers. Predicting the need for the additional staining and trigger it automatically would lead to shorter turn-around times. Thus, the AI prototype was tasked to detect existence of four tumor types. Detailed performance results of the prototype are not the focus here, but results indicate that a majority of IHC orders could indeed be automatically triggered at a feasibly low degree of unnecessary orders.

### Histopathology—Breast Tissue

**Fig. 2:**
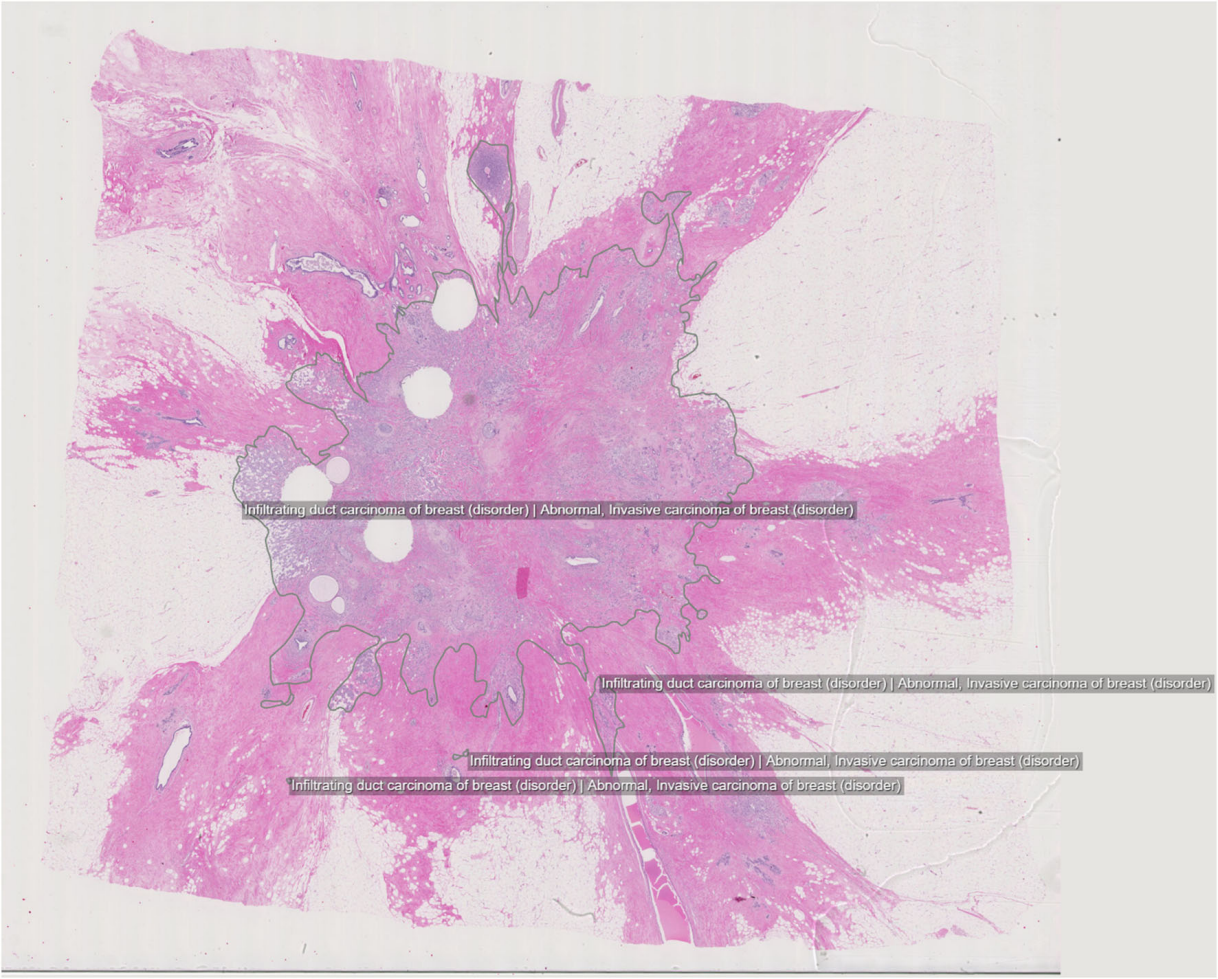
Example image from the DROID breast histopathology dataset, consisting mainly of malignant cases with invasive breast cancer or a mix of invasive and in situ cancer. Full-slide overview with annotated regions

The DROID dataset of breast tissue [16] consists of 361 H&E-stained breast WSI (Fig. 2). Out of these, 296 are malignant cases sampled from women diagnosed with invasive breast cancer or a mix of invasive and in situ cancer. The tumors were classified into four distinct SNOMED-CT categories according to morphology: invasive duct carcinoma, invasive lobular carcinoma, non-invasive in situ carcinoma, and others. In total, 4144 separate annotations were made on the malignant slides to segment the different tissue structures and link them to ontological information. The benign WSIs do not contain any annotations.

The annotation of this breast dataset was also connected to prototype development of an AI application. The target was to provide diagnostic assistance in several ways: per-slide flagging of cancer vs normal, segmenting the cancer tissue within a slide, and finding the greatest tumor diameter. The tumor diameter is a prognostic factor, commonly used by pathologists in their tumor classification work-up. The results from this pilot effort indicate that precision at useful levels is within reach, for example, showing average error margins for tumor segmentation of 0.7 mm.

### Histopathology—Skin Tissue

**Fig. 3:**
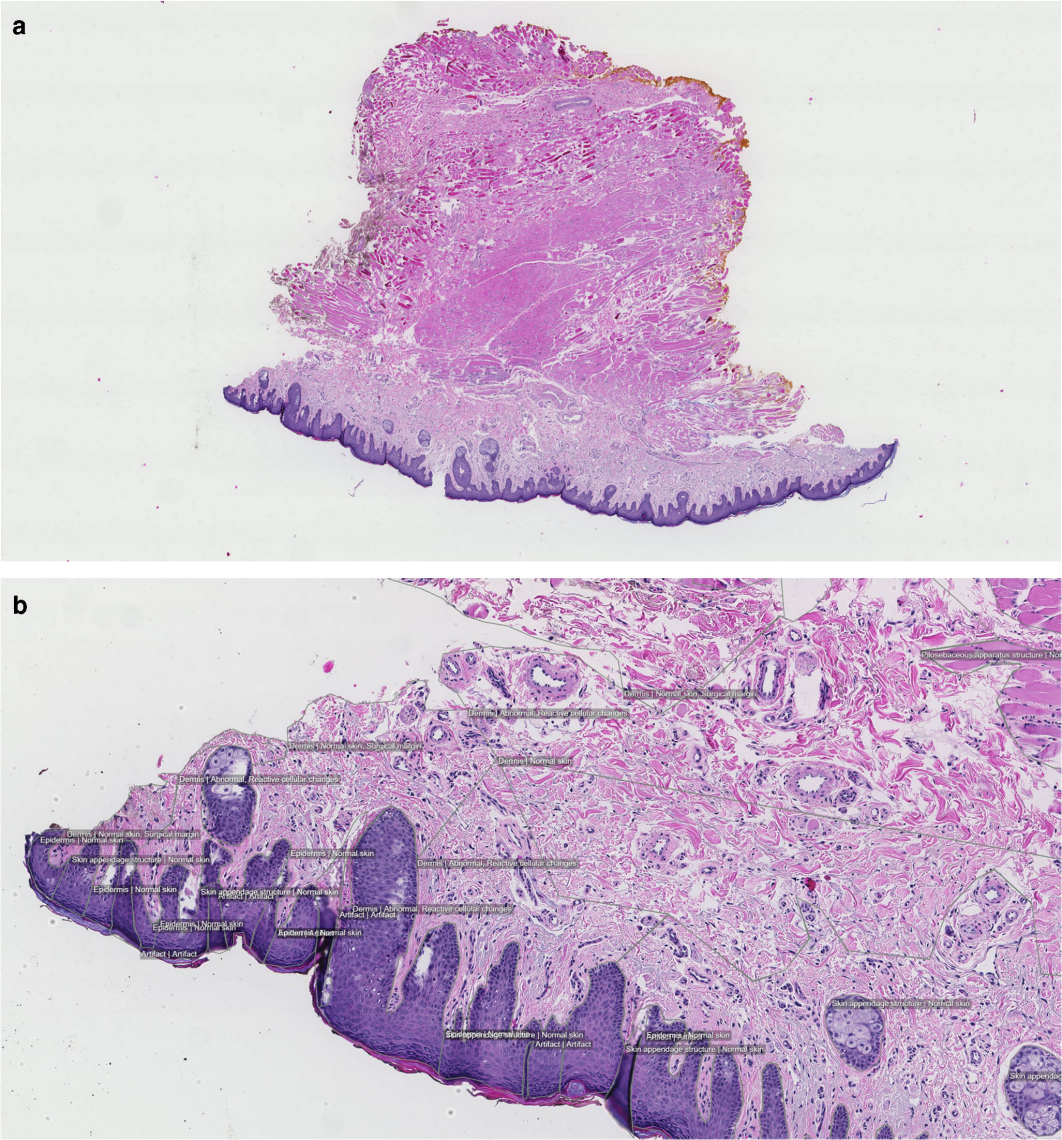
Example images from the DROID skin histopathology dataset, a balanced mix of normal and abnormal cases, with granular abnormality annotations. (**a**) Full-slide overview. (**b**) Close-up showing annotated regions

The WSI collection of skin tissue [17] consists of 99 H&E-stained slides (Fig. 3). Of those, 49 have abnormal features, and 50 are considered histologically normal. For the skin data, a different annotation strategy compared with breast and ovary was applied. All significant abnormal findings were identified and outlined, in total, 13 types such as actinic keratosis, basal cell carcinoma, and dermatofibroma. Other tissue components, such as epidermis and adnexal structures, were also delineated to create a complete histological map, as well as the surgical margin. In total, 16,741 separate annotations were made, all in the form of drawn polygons with ontological labels.

### Histopathology—Colon Tissue

**Fig. 4:**
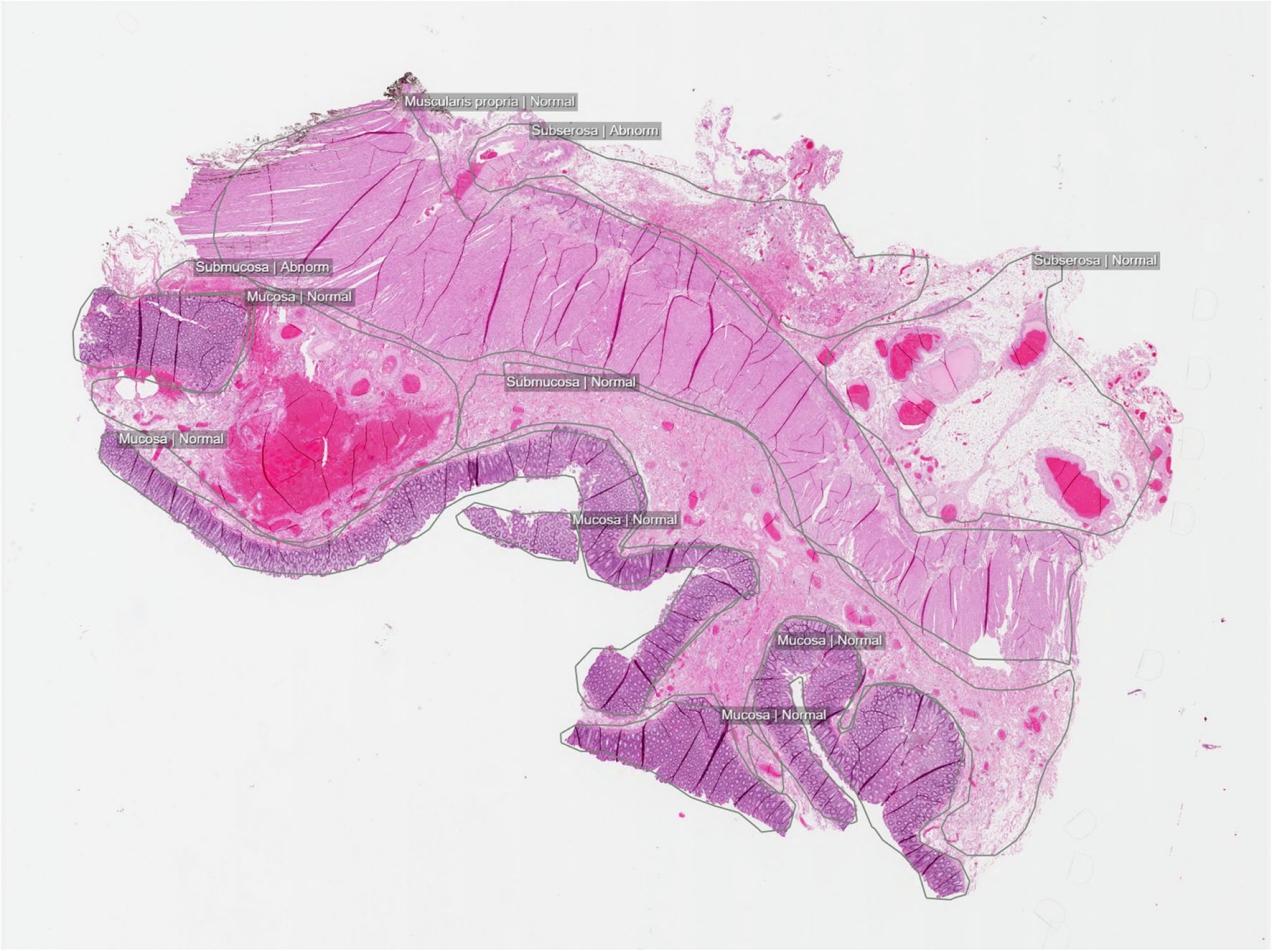
Example image from the DROID colon histopathology dataset, a balanced mix of normal and abnormal cases, with granular abnormality annotations. Full-slide overview with annotated regions

The WSI collection of colon tissue [18] consists of 101 H&E-stained slides (Fig. 4). Of those, 52 have abnormal features, and 49 are considered histologically normal. For the colon data, a similar annotation strategy compared with the skin dataset was applied. All significant abnormal findings were identified and outlined, in total, 15 types such as hyperplastic polyp, high-grade adenocarcinoma and necrosis. Tissue components such as mucosa and submucosa were also delineated to create a complete histological map, as well as the surgical margin. In total, 756 separate annotations were made, all in the form of drawn polygons with ontological labels.

### Radiology—Liver

**Fig. 5:**
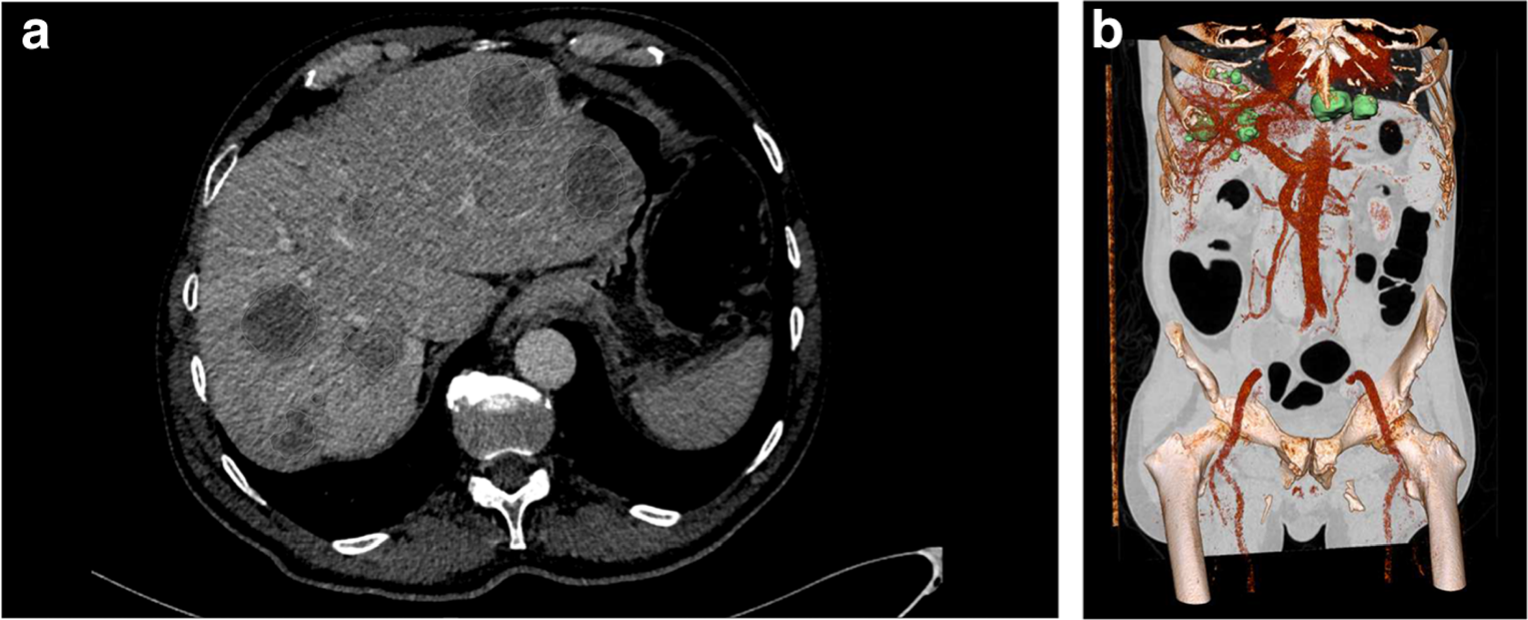
Example images from the DROID liver radiology dataset. (**a**) Axial CT slice of the upper abdomen with seven semi-automated segmented lesions. (**b**) Volume visualization of the whole abdomen with several segmented liver lesions in green. The bones and the portal vein are represented in 3D rendering mode for anatomical orientation. The grayscale image is presented on one coronal slice

The DROID dataset of radiology liver images [19] consists of 76 cases of CT abdomen examinations showing liver metastasis (Fig. 5). The images were acquired in the venous contrast phase with a slice thickness of 1 mm without overlap. All cases included liver malignancies. Multiplanar reconstruction of the CT images was performed to assist localization of the lesions. All lesions > 5 mm were segmented. Obvious cysts were excluded as judged by their Hounsfield Unit pixel values (HU = 0–20). The lesion outlines were done on axial images. The 317 lesions identified were delineated in all slices where they were visible.

The dataset was used in an exploratory AI prototype development effort focused on detecting and segmenting lesions, with each slice evaluated separately. Such computer assistance is desirable as it could lead to time savings for the frequent task to follow up oncology patients [20]. The prototype was developed to a high sensitivity for lesion detection of lesions but with remaining challenges in false-positive rate and segmentation precision.

### Radiology—Skeletal

**Fig. 6:**
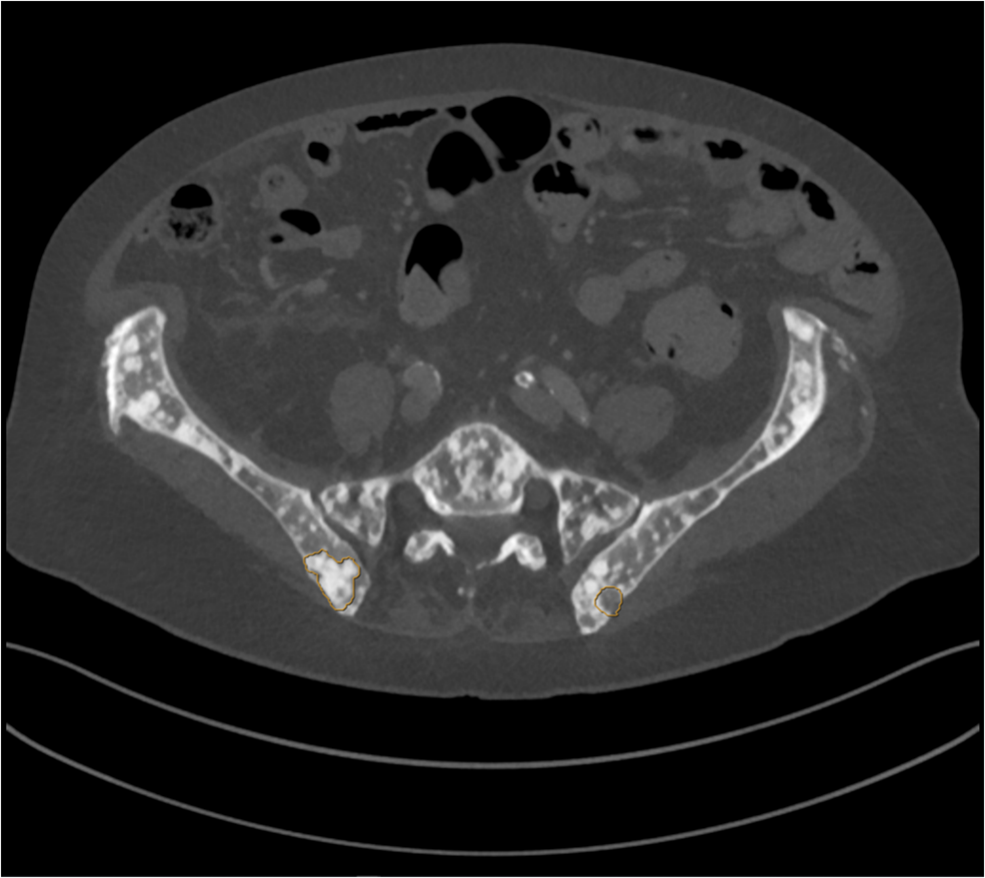
Example image from the DROID skeletal radiology dataset. Axial CT slice with two segmented lesions. On the right side of the patient, one sclerotic lesion is segmented. On the left side of the patient, one lytic lesion is segmented. Observe that the whole pelvic bone is disseminated by both sclerotic and lytic bone metastases

The sixth and smallest oncology dataset in DROID consists of 34 radiology cases of abdominal CT [21]. The scans all include skeletal metastases of lytic or mixed lytic and sclerotic metastases (Fig. 6). Annotations were made for metastasis that had been verified by pathology examination of biopsies. The metastases were given slice-by-slice polygonal outlines by one radiologist, and the annotations were then confirmed by a second radiologist. The dataset contains 36 lesion annotations in total.

### Guiding Principles

In the course of the DROID project, we explored and evaluated the best practices for this type of annotated database construction. The resulting summary of guiding principles is presented in Table 2.

**Table 2:** Guiding principles for proactive database construction

ID	Area	Guiding principle
T1	Team	Ensure rich communication between medical and technical expertise
T2	Team	Promote exchanges across medical subspecialties
P1	Process	Adopt an agile project approach
P2	Process	Use pilot AI development as validation strategy
E1	Execution	Invest in detailed specification of imaging material
E2	Execution	Invest in annotation consistency
E3	Execution	Invest in the development of an annotation strategy
E4	Execution	Invest in customized annotation tools

Principle T1 emphasizes that a cross-disciplinary team with both medical and technical expertise is a prerequisite for a successful outcome. In the concept of medical expertise, we include knowledge on relevant clinical practices. The interdisciplinary interaction is characterized as a recurrent communication with joint incentive to make progress, not a one-off superficial advisory session. Another type of cross-disciplinary is reflected in T2, which highlights the great benefit of involving several diagnostic applications in the same project.

The following two guiding principles apply to process aspects. P1 states that a flexible and iterative approach is to be preferred as opposed to adhering closely to a plan established at the beginning of the effort. The P2 principle means that even when the goal is to produce a database, and explicitly not to develop AI solutions, it is nevertheless advantageous to include AI development to provide feedback on imaging and annotation protocols. An annotation protocol includes many choices such as granularity of categories, type of annotations, and level of detail of delineations. Actual prototype implementations can be used to evaluate whether those choices are appropriate, which can lead to early protocol adjustments to ensure that the time is spent on the most valuable input.

Finally, the guiding principles include four points on how to prioritize efforts. E1–E4 denote aspects that are easily given too little attention and effort. Although E1, specifying imaging material, may sound straightforward, we emphasize the complexities of defining exact inclusion criteria for case types and technical imaging properties. Such criteria also have a close interplay with clinical workflows. The E2 principle refers to the important groundwork on selecting appropriate, standard ontologies for the annotation work. Being closely related, E3 is about specifying what components to annotate, with what precision and exhaustiveness, as well as how to handle overlaps, borderline cases, artifacts, etc. Both E2 and E3 include investment efforts to ensure that the execution takes place according to plan, for instance, through discussion and supervision activities. E4 reflects the insight that even when basic annotation tools such as polygon drawing is sufficient for the task, investing in developing specialized tools can save time as annotation work typically is highly time-consuming.

## Discussion

The presented guiding principles for database creation efforts stem from our experiences in the DROID project. Some of them are conclusions from positive outcomes, whereas others are elicited from obstacles encountered.

The benefit of establishing a cross-disciplinary team, as principle T1 suggests, has been apparent in many situations throughout the project. Essentially, every annotation decision, from low-resolution features such as which organs to target to high-resolution details on how to define delineation for fuzzy boundaries, has required input from both medical and technical expertise. Physicians have been surprised to learn what AI developers consider to be difficult or easy problems to solve, and the technical experts have in turn discovered the many facets of seemingly straightforward clinical decisions. Moreover, we have experienced that the differences between academic and clinical agendas within the medical domain affect the priorities for data collection and annotation. At cross-roads, we have let substantial clinical workload trump directions that have been more intriguing. With regard to T2, we have also found it valuable to discuss difficulties and insights across subprojects. Especially in the histopathology efforts, obstacles that arose for one dataset were often relevant to consider for another as well, such as which level of precision that was appropriate for the different tissue types.

That an agile approach is preferable (P1) is perhaps not surprising but should not be underrated. We have clearly seen that the quality of the data has iteratively increased as initial plans and strategies have been refined. This also links to the need of recurrent communication reflected in T1. The rationale for the P2 principle, to use AI prototyping to provide feedback to the data collection, has been strengthened during our effort. The value of annotations is in our context determined by the usefulness for the AI development. We have experienced concept evolution effect in machine learning [22], i.e., that by developing AI prototypes in parallel to the annotation effort, issues and improvement possibilities for the annotation strategy have been identified. One example is mucinous cancer in the ovarian dataset. In such diseased tissue, mucin is considered to be a part of the tumor, whereas it is a healthy histology component otherwise. Both the annotation and AI development strategies were refined to handle this duality. Conversely, for the liver dataset, it was concluded from the prototyping that addition of a simple bounding box for the liver would have been very helpful, but at that time, annotation time resources were no longer available.

However, for proactive database construction, there is a fundamental and difficult challenge in that all the future potential AI applications are obviously not known. Thus, any validation effort would be incomplete. Nevertheless, there are mitigation strategies. One strategy is to gather broad AI development expertise (compare T1), so that general experience of AI approaches can be considered even if explicit prototypes are built. Another is to plan for refinement and extension of annotations as new needs arise (compare P1).

The reason for including E1, specifying imaging material, as a focus area is simply the many intricate discussions we have had on this topic. As for clinical studies, case inclusion criteria are vital. In addition, decisions as which sequences or immunohistochemistry staining to include are important questions to deliberate. Image resolution, reconstruction parameters, and diversity of scanner equipment are among the technical considerations. Standard ontologies (E2) are a prerequisite for reuse of annotated data, which is further discussed in [14]. In the ovary subproject, the successive refinement of the annotation protocol (E3) concerned outline precision, where the annotator first used an unnecessarily low error tolerance that did not benefit AI training but caused low productivity. The ovary annotation is also an example of benefit of investing in specialized tools (E4). A lesson learned is that time would have been better spent by first developing a highly automated stroma vs epithelium classifier, allowing the manual effort to focus on the epithelial sub-compartments. This type of approach harmonizes very well with P2, as an initial AI prototype for coarse annotation is likely to be a wise path for the more advanced AI development on the same data. Moreover, the benefit of tailor-made annotation tools with the interaction designed to utilize machine learning assistance was demonstrated by positive results from the parallel prototyping effort of the TissueWand tool [11].

Our hope is that this annotated imaging database in its current form will become a valuable steppingstone for future medical AI research. Notably, the pathology datasets are already very mature and rich annotation wise. While the data collections are substantial contributions in relation to currently available data, it is also evident that expansion on a larger scale is a high priority. This is a community effort to which we strive to continue to contribute through our future work.

## Conclusions

Several insights have been made regarding construction of annotated imaging data collections for machine learning. The main points for future similar efforts to consider are that extensive communication in the cross-disciplinary team is essential, an iterative and agile approach is beneficial, and investing in careful preparation is a success factor.

## Appendix

### Pathology

For the pathology images, the annotations were saved separately from the whole-slide images and are available in a standardized format (DICOM CSPS). In all figures, the annotations are rendered on top of the original images. All renderings were made using Sectra PACS 22.1.0.4548 (Sectra AB, Sweden).

#### Ovarian Tissue

The DROID dataset consists of 193 HE stained, anonymized ovary WSI collected from women of an age range of 17–86 years. The overall distribution of diagnostic classifications was assessed to reflect the overall distribution of clinical cases of suspected ovarian tumors over the time period of 2015–2017. This includes 172 malignant tumor cases sampled from women in the age range 17–86 years and 21 normal cases from women in the age range 51–75 years. The WSIs were scanned on a Scanscope AT (Aperio, US), a NanoZoomer XR (Hamamatsu, Japan), or a NanoZoomer XRL (Hamamatsu, Japan). Eight of the most prevalent, histological defined, tumor types were chosen to be annotated: high-grade serous carcinoma (HGSC), low-grade serous carcinoma (LGSC), clear-cell carcinoma (CC), endometrioid adenocarcinoma (EN), metastatic serous carcinoma (MS), serous borderline tumor (SB), mucinous borderline tumor (MB), and metastatic adenocarcinoma (MO). In total, 2402 separate annotations were made to define the different tissue structures. Normal ovarian tissue was outlined as a whole.

#### Breast Tissue

The DROID dataset consists of 361 HE-stained, anonymized breast WSI. Out of these, 296 are malignant images sampled from women diagnosed with invasive breast cancer or a mix of invasive and in situ cancer. The WSI were all scanned on a NanoZoomer (Hamamatsu, Japan): XR C12000 series 2013 or a 2.0 HT C9600 series 2013. The dataset also contains 65 benign breast images scanned on a NanoZoomer 2.0 HT C9600 series 2013. The tumors were classified into four distinct SNOMED-CT categories upon morphology: invasive duct carcinoma, invasive lobular carcinoma, non-invasive in situ carcinoma, and others. In total, 4144 separate annotations were made on the malignant WSI to segment the different tissue structures and link them to ontological information. The benign WSI does not yet have any annotations.

#### Skin Tissue

The DROID dataset consists of 99 HE-stained, anonymized skin WSI. 49 skin WSI show abnormal features sampled from male and females in the age range of 29–93 years. Furthermore, 50 normal skin WSI were sampled from males and females in an age range of 20–93 years. All skin WSI were scanned on a Scanscope AT (Aperio, US), a NanoZoomer XR (Hamamatsu, Japan), or a NanoZoomer XRL (Hamamatsu, Japan). Careful detailed annotations were made to segment all abnormal findings identified, i.e., actinic keratosis, basal cell carcinoma, dermatofibroma, dysplastic nevus, intradermal nevus, keratoacanthoma, lentigo malignant melanoma, malignant melanoma, malignant melanoma in situ, scar, seborrheic keratosis, squamous cell carcinoma, and squamous cell carcinoma in situ. Other areas annotated are abnormal, acanthosis, artifacts, dermis, epidermis, fibrosis, fibrin body, granuloma, inflammation, inflammatory edema, normal, perichondrium, reactive cellular changes, skin appendage structure, surgical margins, structure of cartilage of auditory canal, subcutaneous fatty tissue, subcutaneous tissue, and from which body part the skin was excised. In the normal skin cases, the following structures were annotated: artifact, dermis, epidermis, normal skin, perichondrium, skin and subcutaneous structure, skin appendage structure, skin structure, structure of cartilage of auditory canal, subcutaneous fatty tissue, subcutaneous tissue, and surgical margins. In total, 16,741 separate annotations were made on the WSI to segment the different tissue structures and link them to ontological information.

#### Colon Tissue

The DROID dataset consists of 101 HE-stained, anonymized colon WSI. All colon WSI were scanned with Scanscope AT (Aperio, US), NanoZoomer XR (Hamamatsu, Japan), or NanoZoomer XRL (Hamamatsu, Japan). 52 abnormal colon cases were collected from male and females in an age range of 22–90 years. These WSI were detailed annotated, and the following structures were labeled: acute and chronic inflammation, acute inflammation, adenocarcinoma, atrophy, chronic inflammation, diverticula, diverticulitis, dysplasia, edema, fibrosis, granulation tissue, hemorrhage, hyalinization, hyperplasia, hyperplastic polyp, inflammation, lymphoma, mucinous adenocarcinoma, necrosis, serrated adenoma, stasis, tubular adenoma, tubulovillous adenoma, and ulceration. Other areas annotated were abnormal, artifact, cecum, colon, colonic mucous membrane, colonic muscularis propria, colonic submucosa, colonic subserosa, descending colon, ileum, normal, rectum, sigmoid colon, and transverse colon. Furthermore, 49 normal WSI sampled from the same age span were annotated to label the following structures: artifact, cecum, colon, colonic mucous membrane, colonic muscularis propria, colonic submucosa, colonic subserosa, descending colon, ileum, normal, rectum, sigmoid colon, and transverse colon. In total, 756 separate annotations were made according to a similar, very detailed, annotation strategy as for the skin.

### Radiology

For the radiology images, the segmentations were saved separately from the axial radiology images and are available in a standardized format (DICOM SEG and DICOM GSPS). All 2D renderings were made using Sectra PACS 22.1.0.4548 (Sectra AB, Sweden) apart from the 3D rendering, which was made using Slicer 4.10.2 r28257.

#### Liver Tissue Radiology Images

The DROID dataset consists of 76 cases of CT abdomen examinations showing liver metastasis. All datasets were performed in the venous contrast phase with a slice thickness of 1 mm and no overlap. All cases included showed liver malignancies. Lesions > 5 mm were segmented with a custom-made semi-automatic segmentation tool integrated with the Sectra PACS system with a pen on a tablet computer. When delineating a lesion with the pen, the software would compute a probable segmentation based on the areas indicated by the user simply by drawing a line across the lesion. This helped in increasing the speed of the delineation process. The suggested delineation was then adjusted slice by slice on the axial images. From the sum of all segmentations, a volume was generated. Obvious benign liver cysts were excluded as defined by Hounsfield Units (HU = 0–20). In total, 317 lesions were annotated. The modality models were mainly Siemens SOMATOM Definition AS/AS+, but other Siemens SOMATOM models were also included: Force, Drive, and Definition Edge.

#### Skeletal Metastasis Radiology Images

The DROID dataset consists of 34 radiology cases of CT abdomen showing lytic (bone degrading) and mixed lytic/sclerotic metastasis from prostate, breast, and lung primary cancer. Annotations were made for biopsied metastasis that had been pathologically proven as malignancies. The data contains 36 lesion annotations in total. The same annotation process as for the liver data was used. The modality models were mainly Siemens SOMATOM Definition AS/AS+, but several other models were also included: Siemens SOMATOM models Force, Drive, and Definition Edge, Philips Ingenuity Core, and GE Discovery CT750 HD.
